# Prognostic Value of the Objective Prognostic Score and Palliative Prognostic Index for Short-Term Mortality in Terminal Cancer Patients Receiving Best Supportive Care: A Prospective Observational Single-Center Study

**DOI:** 10.3390/jcm15124502

**Published:** 2026-06-10

**Authors:** Alperen Akansel Çağlar, Zekeriya Hannarici, Mehmet Emin Büyükbayram, Aykut Turhan, Yasin Emrah Soylu, Mehmet Bilici, Salim Başol Tekin

**Affiliations:** 1 Department of Medical Oncology, Başakşehir Çam and Sakura City Hospital, Istanbul 34110, Turkey; 2 Department of Medical Oncology, Bursa Yuksek Ihtisas Training and Research Hospital, Bursa 16010, Turkey; hannarici@hotmail.com; 3 Department of Medical Oncology, Yalova State Hospital, Yalova 77100, Turkey; m.eminbuyukbayram@hotmail.com; 4 Department of Medical Oncology, Ordu University Training and Research Hospital, Ordu 52200, Turkey; dr.aykutturhan@gmail.com; 5 Department of Medical Oncology, Training and Research Hospital, Faculty of Medicine, Atatürk University, Erzurum 25030, Turkey; y.e.s138@hotmail.com (Y.E.S.); drmbilici@gmail.com (M.B.); 6 Department of Medical Oncology, Acibadem Bursa Hospital, Bursa 16010, Turkey; salim_b_tekin@hotmail.com

**Keywords:** palliative prognostic index, objective prognostic score, terminal cancer, best supportive care, prognostication, palliative care

## Abstract

**Background**: Accurate prognostication in terminal cancer patients receiving best supportive care (BSC) is essential for guiding end-of-life decision-making and avoiding non-beneficial interventions. Several prognostic models have been developed for advanced cancer, including the Palliative Prognostic Index (PPI) and the Objective Prognostic Score (OPS). However, prospective data evaluating their performance specifically in patients managed with BSC are limited. This study evaluated the prognostic performance of PPI and OPS in terminal cancer patients receiving BSC. It also examined whether their combined use provides additional value for short-term mortality risk stratification. **Methods**: This prospective observational cohort study included hospitalized adult patients with terminal-stage cancer and a documented BSC decision. Terminal-stage cancer was operationally defined as stage IV malignancy with poor performance status and no remaining feasible disease-directed oncological treatment option due to severe clinical deterioration and/or major organ dysfunction. Patients were prospectively enrolled from 12 April 2024 to 13 December 2024 and followed until death. Eligible patients had poor Eastern Cooperative Oncology Group performance status (ECOG 3–4) and had not received oncologic treatment within the preceding month. PPI and OPS were calculated at baseline using predefined criteria. Survival time was defined as the interval between baseline assessment and death. The prognostic performance of the scores for 3-, 4-, and 6-week mortality was evaluated, and survival outcomes were analyzed using standard survival analysis methods. **Results**: A total of 112 patients were included in the final analysis. The mean age was 62.3 ± 12.3 years; 66 patients (58.9%) were male and 46 (41.1%) were female. The most common primary tumor sites were colon cancer (20.5%), non-small cell lung cancer (17.0%), and gastric cancer (15.2%). Both PPI > 6 and OPS ≥ 3 were associated with higher short-term mortality, although their individual discriminatory performance was modest. The combined OPS–PPI approach showed statistically significant but still modest discrimination at all time points. Although this difference was limited, the combined approach supported the stratification of a clinically relevant subgroup at particularly high risk of imminent death. Patients with both OPS ≥ 3 and PPI > 6 had the poorest survival, with a median overall survival (OS) of 11 days. In multivariable Cox regression analysis, the combined high-risk group remained independently associated with poorer OS (HR 1.53, 95% CI 1.01–2.31; *p* = 0.046). **Conclusions**: Although the individual discriminatory performance of PPI and OPS was modest, their combined use may provide additional risk stratification value and may help identify patients at particularly high risk of short-term mortality among terminal cancer patients receiving BSC. These findings should be interpreted as supporting bedside risk stratification rather than indicating a definitive individual-level prediction model.

## 1. Introduction

Advanced cancer is generally defined as metastatic, locally advanced, or recurrent malignant disease that is no longer amenable to curative treatment and is commonly characterized by a progressive clinical course, high symptom burden, functional decline, and limited life expectancy [1]. The global cancer burden remains substantial; according to recent estimates, approximately 20 million new cancer cases and 9.7 million cancer-related deaths occurred worldwide in 2022 [2]. Accordingly, the need for palliative and end-of-life care among patients with advanced and terminal-stage cancer is becoming increasingly important. Because patients with terminal-phase advanced cancer frequently experience rapid clinical deterioration, accurate prognostic assessment represents a fundamental component of high-quality palliative care. Reliable prognostic assessment may facilitate timely best supportive care (BSC) decisions, avoidance of non-beneficial interventions, appropriate referral to palliative care services, and realistic communication with patients and their families [1]. Nevertheless, previous studies have shown that clinicians often overestimate survival in patients with terminal cancer, which may lead to delayed end-of-life discussions and potentially non-beneficial treatments [3,4,5].

To address this challenge, several prognostic models have been developed specifically for patients with advanced or terminal cancer. Among these, the Palliative Prognostic Index (PPI) is one of the most widely used and validated tools in palliative care settings [6]. The PPI is a clinical prognostic index composed of five parameters: Palliative Performance Scale (PPS), oral intake, presence of edema, dyspnea at rest, and delirium. Each component is assigned a weighted score, with higher total scores indicating poorer prognosis. Previous studies, recent systematic reviews, and contemporary validation studies have shown that PPI-based assessments are useful for short-term survival estimation, especially within 3 to 6 weeks, and that higher PPI scores are associated with shorter survival [6,7,8,9,10].

Despite its robust validation, PPI incorporates clinical assessments such as performance status, oral intake, and delirium, which may be subject to interobserver variability, especially across different care settings and levels of clinician experience [11]. This limitation has stimulated interest in prognostic tools based exclusively on objectively measurable variables.

The Objective Prognostic Score (OPS) was developed as a prognostic model primarily based on objective clinical and laboratory parameters, minimizing reliance on subjective clinical judgment [12]. OPS includes eight variables: Eastern Cooperative Oncology Group performance status (ECOG PS = 4), anorexia (defined as intake of fewer than five spoonfuls per meal or less than one-third of a normal meal), dyspnea at rest, elevated white blood cell (WBC) count (>11,000/μL), hyperbilirubinemia (total bilirubin > 2.0 mg/dL), renal dysfunction (serum creatinine ≥ 1.5 mg/dL), and elevated lactate dehydrogenase (LDH ≥ 502 IU/L). Renal dysfunction (serum creatinine ≥ 1.5 mg/dL) is assigned 2 points, whereas each of the remaining parameters contributes 1 point to the total score. Higher OPS values are associated with poorer prognosis and shorter expected survival [12,13,14,15].

Comparative studies evaluating PPI, OPS, and other prognostic models suggest that these tools demonstrate broadly comparable prognostic performance, while differing in complexity, objectivity, and clinical applicability [11,13,16,17,18]. However, most existing studies have been retrospective in design, conducted in heterogeneous palliative care populations, or have included patients receiving mixed treatment intents. Furthermore, although some prospective studies have compared PPI and OPS within the same patient cohorts, they have primarily focused on the individual prognostic performance of each score rather than evaluating their structured integration as a combined risk stratification approach. Importantly, evidence derived from prospective cohorts specifically limited to terminal cancer patients for whom a BSC decision has been made remains scarce. Therefore, prospective data on the combined prognostic use of PPI and OPS in a homogeneous BSC cohort remain limited [19]. The combined assessment of PPI and OPS may be clinically meaningful because these two scores capture different but complementary dimensions of terminal deterioration. While PPI mainly reflects clinical and functional decline, OPS provides a more objective assessment of physiological and laboratory-based deterioration. Therefore, combining these tools may offer a broader prognostic perspective than either score alone and may support the structured identification of patients at high risk of short-term mortality.

Given these gaps, the present study was designed to assess the individual prognostic performance of PPI and OPS and to explore whether their combined use provides additional value for short-term mortality risk stratification in terminal cancer patients receiving BSC.

## 2. Study Aim

This prospective observational study aimed to assess the individual prognostic value of the PPI and OPS for short-term mortality risk stratification in terminal cancer patients managed with BSC. It also evaluated whether their combined use provides additional risk stratification value in identifying patients at high risk of short-term mortality.

## 3. Materials and Methods

### 3.1. Study Design and Patient Population

This prospective observational study was conducted in a tertiary-level comprehensive cancer center. Hospitalized adult patients with advanced cancer were screened for eligibility.

Terminal-stage cancer was operationally defined as stage IV malignancy with poor performance status and no remaining feasible disease-directed oncological treatment option due to severe clinical deterioration and/or major organ dysfunction, including hepatic, renal, cardiac, or bone marrow failure. Poor performance status was defined as ECOG PS 3 or 4. ECOG PS 3 indicated that the patient was capable of only limited self-care and confined to bed or chair for more than 50% of waking hours, whereas ECOG PS 4 indicated that the patient was completely disabled, unable to carry out any self-care, and totally confined to bed or chair.

The decision for BSC was made by the medical oncology clinical council according to a standardized institutional approach. This decision was based on the absence of expected meaningful clinical benefit from further chemotherapy, targeted therapy, immunotherapy, or radiotherapy; ECOG PS 3 or 4; and the presence of advanced organ dysfunction or clinical deterioration precluding further anticancer treatment.

Patients were included if they met all of the following criteria: (1) stage IV cancer, (2) a documented decision for BSC determined by the medical oncology clinical council, (3) no receipt of any oncological treatment, including chemotherapy, targeted therapy, immunotherapy, or radiotherapy, within the preceding one month, and (4) poor performance status defined as ECOG PS 3 or 4.

To minimize selection bias, all eligible patients hospitalized in the medical oncology ward during the study period were prospectively and consecutively enrolled.

### 3.2. Data Collection and Prognostic Score Assessment

All included patients underwent a comprehensive clinical evaluation on the first day of hospital admission. Physical examination findings and laboratory parameters were recorded at baseline by the responsible investigator. To reduce inter-rater variability, all baseline clinical assessments were performed by the responsible investigator, and OPS and PPI scores were calculated by the same investigator. On the same day, the PPI and the OPS were calculated for each patient according to their original definitions.

PPI was calculated based on five clinical variables: PPS, oral intake, presence of edema, dyspnea at rest, and delirium. OPS was calculated using objective clinical and laboratory parameters, including ECOG performance status, anorexia, dyspnea at rest, WBC count, total bilirubin, serum creatinine, and LDH levels. The detailed scoring criteria for OPS, PPI, and PPS are provided in Supplementary Tables S1, S2, and S3, respectively.

### 3.3. Follow-Up and Outcome Measures

A total of 114 consecutive patients were prospectively enrolled between 12 April 2024 and 13 December 2024. Patients were followed until death, with data collection continuing until the date of death of the last patient, 5 May 2025. Survival time was defined as the interval between the date of hospital admission, corresponding to baseline assessment, and the date of death.

Clinical outcome data were unavailable for two patients during follow-up. Therefore, the final analysis included 112 patients. These patients were excluded using a complete-case analysis approach, and no imputation was performed because survival outcome data were unavailable.

### 3.4. Ethics Approval and Informed Consent

This prospective observational study was conducted in accordance with the principles of the Declaration of Helsinki. Ethical approval was obtained from the Atatürk University Non-Interventional Clinical Research Ethics Committee with approval number B.30.2.ATA.0.01.00/529, dated 27 September 2024. Written informed consent was obtained from all participants before enrollment. Given the vulnerable nature of the study population, the informed consent process was conducted with particular attention to patients’ clinical condition, decision-making capacity, and respect for patient autonomy. No interventional procedure or treatment allocation was performed as part of the study.

### 3.5. Statistical Analysis

An a priori sample size calculation was performed using G*Power version 3.1.9.7 (Heinrich Heine University Düsseldorf, Germany) before patient enrollment. The calculation was based on logistic regression analysis for short-term mortality prediction. Assuming a two-sided alpha level of 0.05, 80% statistical power, an odds ratio of 4.0 for high-risk prognostic score groups, Pr(Y = 1|X = 1) under H0 of 0.50, R^2^ with other covariates of 0.20, and a binomial distribution of the predictor with π = 0.50, the minimum required total sample size was calculated as 101 patients. Therefore, the final analyzed cohort of 112 patients was considered sufficient for the primary statistical analysis.

Statistical analyses were performed using IBM SPSS Statistics for Windows, Version 25.0 (IBM Corp. Armonk, NY, USA). Descriptive statistics were expressed as number and percentage for categorical variables, and as mean ± standard deviation or median (minimum–maximum) for continuous variables, as appropriate.

PPI and OPS were primarily analyzed as dichotomized variables using predefined cut-off values, PPI > 6 and OPS ≥ 3, because these thresholds have been used in previous validation studies and provide clinically practical risk categories for bedside decision-making in palliative care. This approach was chosen to facilitate interpretation and clinical applicability in terminal cancer patients receiving BSC.

The discriminatory performance of clinical variables for short-term mortality was evaluated using receiver operating characteristic (ROC) curve analysis. Pairwise comparisons of area under the curve (AUC) values between OPS, PPI, and the combined OPS–PPI model were performed using the DeLong test.

Model calibration was assessed using Brier scores for OPS, PPI, and the combined OPS–PPI model at 3-, 4-, and 6-week mortality time points. The Hosmer–Lemeshow goodness-of-fit test was also explored; however, because the evaluated prognostic models were based on dichotomized predictors, the test had limited applicability and was not considered informative when degrees of freedom were zero.

Survival outcomes were estimated using the Kaplan–Meier method and compared between groups with the log-rank test. Cox proportional hazards regression analysis was used to evaluate factors associated with overall survival (OS). The proportional hazards assumption for the Cox regression model was assessed graphically using log-minus-log survival plots, and no obvious violation was observed. In addition to analyses based on predefined cut-off values, PPI and OPS were also evaluated as continuous variables in sensitivity ROC and Cox regression analyses. Decision curve analysis was performed to estimate the clinical net benefit of OPS, PPI, and the combined OPS–PPI approach across threshold probabilities for 3-, 4-, and 6-week mortality prediction. A two-sided *p* value of <0.05 was considered statistically significant.

## 4. Results

A total of 114 consecutive patients were prospectively enrolled in the study. Survival outcome data were unavailable for two patients; therefore, the final analysis included 112 patients.

**Figure 1:**
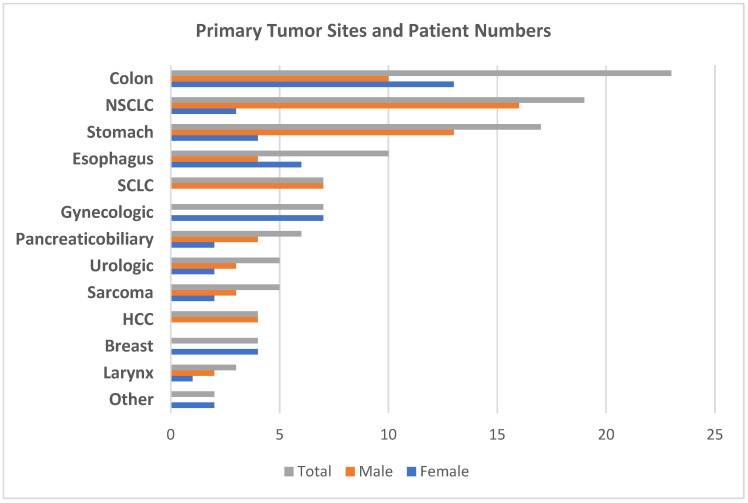
Patient numbers according to primary tumor site. HCC, hepatocellular carcinoma; NSCLC, non-small cell lung cancer; SCLC, small cell lung cancer.

The mean age of the cohort was 62.3 ± 12.3 years (median, 61.5; range, 29–86), and 66 patients (58.9%) were male. The most common primary tumor site was colon cancer (*n* = 23, 20.5%), followed by non-small cell lung cancer (NSCLC; *n* = 19, 17.0%), stomach cancer (*n* = 17, 15.2%), and esophageal cancer (*n* = 10, 8.9%). The distribution of primary tumor sites according to sex is presented in Figure 1.

For risk stratification, patients were categorized using their established cut-off values for the PPI > 6 and the OPS ≥ 3, and an additional combined OPS–PPI risk grouping was constructed. Based on this combined classification, 61 of 112 patients (54.5%) were assigned to the highest-risk category (OPS ≥ 3 and PPI > 6). The remaining baseline sociodemographic and clinical characteristics, as well as short-term mortality outcomes, are summarized in Table 1.

The discriminatory performance of OPS, PPI, and the combined OPS–PPI model for predicting short-term mortality at 3, 4, and 6 weeks is presented in Table 2.

For 3-week mortality, both OPS ≥ 3 and PPI > 6 showed statistically significant but modest discriminatory performance, with identical AUC values of 0.617 (95% CI, 0.514–0.721; *p* = 0.033) and 0.617 (95% CI, 0.512–0.721; *p* = 0.034), respectively. The combined OPS–PPI model demonstrated a numerically higher AUC of 0.674 (95% CI, 0.573–0.774; *p* = 0.002); however, this value also remained below 0.70, indicating modest discriminative ability.

At 4 weeks, OPS ≥ 3 showed borderline discriminatory performance, with an AUC of 0.598 (95% CI, 0.492–0.704; *p* = 0.074), whereas PPI > 6 did not demonstrate statistically significant discrimination, with an AUC of 0.545 (95% CI, 0.437–0.652; *p* = 0.414). The combined OPS–PPI model showed statistically significant but limited discrimination for 4-week mortality, with an AUC of 0.613 (95% CI, 0.508–0.718; *p* = 0.039).

For 6-week mortality, OPS ≥ 3 showed statistically significant but modest discriminatory performance, with an AUC of 0.643 (95% CI, 0.532–0.754; *p* = 0.012), whereas PPI > 6 did not reach statistical significance, with an AUC of 0.578 (95% CI, 0.466–0.690; *p* = 0.174). The combined OPS–PPI model demonstrated statistically significant but still modest discrimination, with an AUC of 0.651 (95% CI, 0.545–0.758; *p* = 0.008).

Overall, although several ROC analyses reached statistical significance, most AUC values remained below 0.70, indicating that the discriminatory performance of OPS, PPI, and the combined OPS–PPI approach was modest. In addition, the relatively wide confidence intervals around some AUC estimates suggest uncertainty related to the limited sample size and should be considered when interpreting discriminatory performance. Therefore, these findings should be interpreted as supporting risk stratification rather than strong individual-level prediction.

Pairwise DeLong comparisons were performed to formally compare the discriminatory performance of OPS, PPI, and the combined OPS–PPI model. No statistically significant difference was observed between OPS and PPI for 3-, 4-, or 6-week mortality prediction (*p* = 0.989, *p* = 0.300, and *p* = 0.253, respectively). Similarly, the AUC of the combined OPS–PPI model was not significantly different from OPS alone at any time point (*p* = 0.141, *p* = 0.696, and *p* = 0.840, respectively). However, the combined OPS–PPI approach demonstrated significantly higher AUC values than PPI alone for 3-, 4-, and 6-week mortality prediction (*p* = 0.018, *p* = 0.010, and *p* = 0.021, respectively). These findings suggest that the combined approach may provide incremental discrimination compared with PPI alone, but not consistently beyond OPS alone.

Calibration was further evaluated using Brier scores. For 3-week mortality prediction, the Brier scores were 0.2301 for OPS, 0.2344 for PPI, and 0.2186 for the combined OPS–PPI model. For 4-week mortality prediction, the corresponding Brier scores were 0.2301, 0.2466, and 0.2359, respectively. For 6-week mortality prediction, the Brier scores were 0.2623 for OPS, 0.2241 for PPI, and 0.2101 for the combined OPS–PPI model. Overall, the combined OPS–PPI model showed comparable or slightly lower Brier scores than the individual models, particularly for 3- and 6-week mortality prediction. The Hosmer–Lemeshow test was explored but was not considered informative because the dichotomized model structures resulted in limited degrees of freedom.

Table 3 presents the comparison of 3-, 4-, and 6-week mortality rates according to the combined OPS–PPI risk groups.

A statistically significant difference in mortality was observed among the groups at all evaluated time points (3-week *p* = 0.002, 4-week *p* = 0.020, and 6-week *p* = 0.002).

The highest mortality consistently occurred in the OPS ≥ 3 and PPI > 6 group, with rates of 62.3% at 3 weeks, 63.9% at 4 weeks, and 77.0% at 6 weeks. The3 and PPI3 & PPI > 6 subgroup included only nine patients and showed lower mortality rates; therefore, this subgroup-level finding should be interpreted cautiously. Detailed mortality distributions are shown in Table 3.

**Figure 2:**
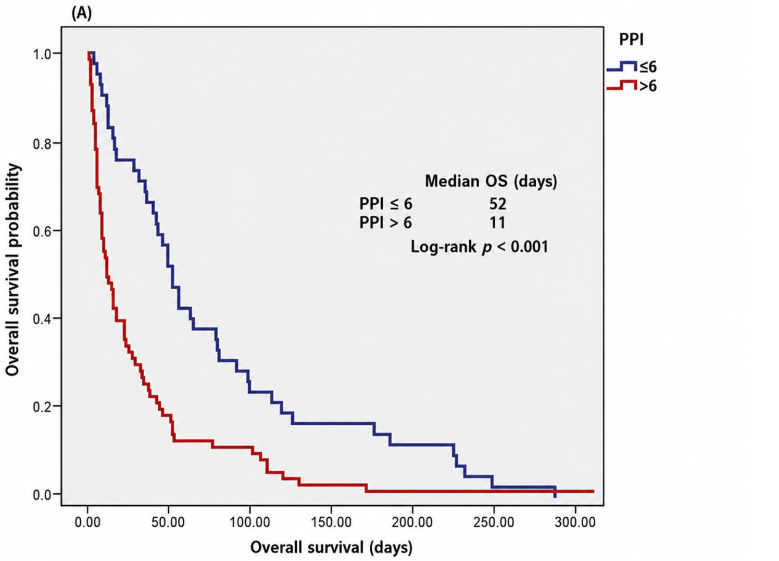

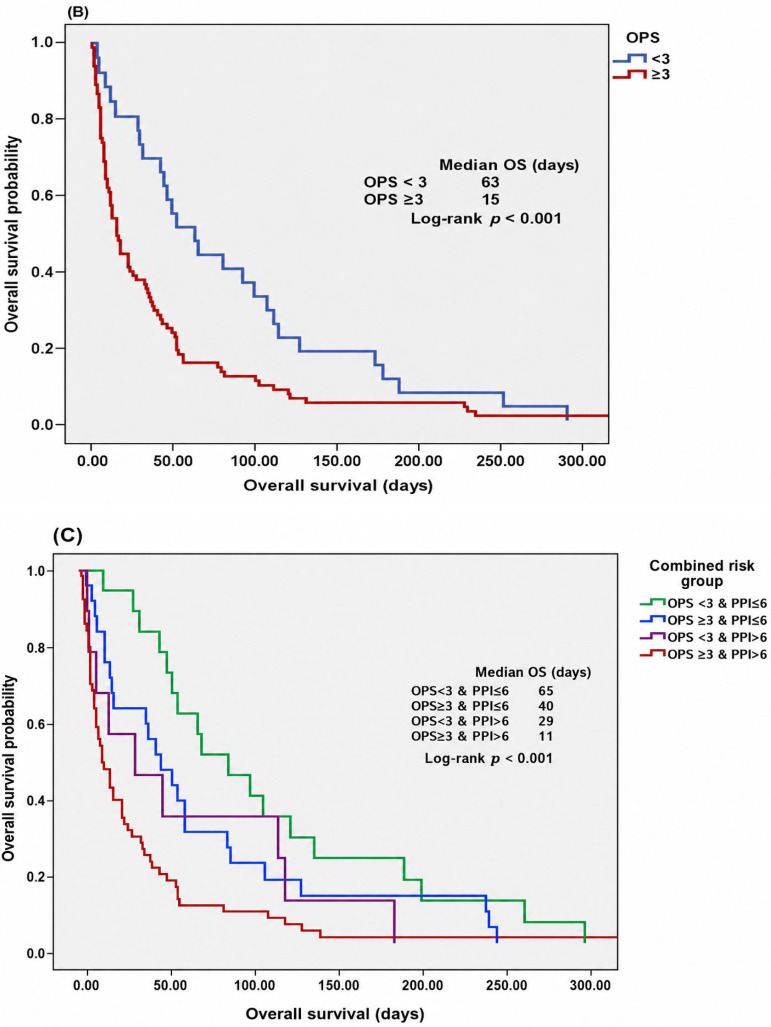
Kaplan–Meier overall survival curves according to PPI, OPS, and combined OPS–PPI risk groups. (**A**) Overall survival according to PPI group (≤6 vs. >6). (**B**) Overall survival according to OPS group (<3 vs. ≥3). (**C**) Overall survival according to the combined OPS–PPI risk groups. Survival curves were compared using the log-rank test. OPS, Objective Prognostic Score; PPI, Palliative Prognostic Index; OS, overall survival.

OS was compared between risk groups using Kaplan–Meier analysis (Figure 2). Patients with higher PPI scores (>6) had significantly shorter median OS than those with PPI ≤ 6 (11 vs. 52 days, *p* < 0.001). Similarly, patients with OPS ≥ 3 demonstrated significantly poorer survival compared with those with OPS < 3 (15 vs. 63 days, *p* < 0.001).

When patients were stratified according to the combined OPS–PPI model, a clear gradient in survival was observed across the four risk categories. The worst survival was seen in patients with both high OPS and high PPI (OPS ≥ 3 and PPI > 6; median OS 11 days), whereas the best survival occurred in patients with both low OPS and low PPI (OPS < 3 and PPI ≤ 6; median OS 65 days) (Figure 2).

Multivariable Cox regression analysis was performed to evaluate the prognostic impact of the combined OPS–PPI high-risk group after adjustment for age, sex, primary tumor site, C-reactive protein (CRP), and albumin (Table 4). The combined high-risk group remained independently associated with poorer OS (HR 1.53, 95% CI 1.01–2.31; *p* = 0.046), and age was also independently associated with survival (HR 1.02, 95% CI 1.00–1.04; *p* = 0.025). In contrast, sex, primary tumor site, CRP, and albumin were not independently associated with OS. Primary tumor site was grouped into major tumor categories to reduce model instability due to sparse tumor-specific subgroups. The model was intentionally limited to non-overlapping clinical variables because several key clinical and laboratory parameters were already incorporated within the PPI and OPSs.

In addition to the predefined cut-off-based analyses, sensitivity analyses were performed using PPI and OPS as continuous variables to assess whether dichotomization materially influenced the findings. In sensitivity ROC analyses, both continuous PPI and continuous OPS showed statistically significant discriminatory performance for 3-week and 6-week mortality, whereas neither score reached statistical significance for 4-week mortality. Continuous PPI showed AUC values of 0.681 for 3-week mortality, 0.599 for 4-week mortality, and 0.641 for 6-week mortality. The corresponding AUC values for continuous OPS were 0.629, 0.586, and 0.655, respectively. These findings were generally consistent with the main cut-off-based analyses, supporting the overall direction of the primary results. The detailed sensitivity ROC results are presented in Table 5.

In Cox regression sensitivity analyses using PPI and OPS as continuous variables, both scores were significantly associated with OS in univariate analyses. For each 1-point increase, PPI was associated with poorer OS (HR 1.08, 95% CI 1.01–1.16; *p* = 0.023), and OPS was also associated with poorer OS (HR 1.15, 95% CI 1.01–1.32; *p* = 0.036). However, after adjustment for age, sex, and primary tumor site, these associations were attenuated and did not remain statistically significant for either PPI (HR 1.06, 95% CI 0.98–1.15; *p* = 0.137) or OPS (HR 1.06, 95% CI 0.90–1.25; *p* = 0.463). These results suggest that the continuous-variable sensitivity analyses did not materially contradict the primary cut-off-based findings, but they also support a cautious interpretation of the independent prognostic contribution of the individual scores. The detailed Cox regression sensitivity results are presented in Table 6.

Decision curve analysis was performed to compare the clinical net benefit of OPS, PPI, and the combined OPS–PPI approach for 3-, 4-, and 6-week mortality prediction. For 3-week mortality, the combined OPS–PPI approach demonstrated higher net benefit than the individual OPS and PPI scores across the evaluated clinically relevant decision thresholds. Similarly, in the 6-week mortality analysis, the combined approach showed higher net benefit than the individual scores. In contrast, for 4-week mortality prediction, the net benefit of the combined OPS–PPI approach was more limited and showed a curve pattern that largely overlapped with those of the individual OPS and PPI scores. Overall, the decision curve analysis findings favored the combined OPS–PPI approach most clearly for 3-week and 6-week mortality prediction, whereas for 4-week mortality prediction the findings were more limited and largely comparable to the individual OPS and PPI scores.

## 5. Discussion

Accurate prognostication in terminal cancer patients is essential for guiding end-of-life decision-making. The PPI is a well-validated clinical tool for predicting short-term survival, particularly within the last weeks of life [6,7,8]. In contrast, the OPS was developed to reduce subjectivity by incorporating measurable laboratory and physiological parameters [12,13]. Recent literature suggests that integrating clinical and objective prognostic indicators may improve risk stratification in advanced cancer patients [13,16]. Prospective data evaluating the complementary or combined prognostic use of PPI and OPS within a homogeneous BSC population remain limited. When analyzed according to predefined OPS and PPI cut-off values, both scores demonstrated statistically significant discriminatory ability for short-term mortality [6,7,8,12,13]. Nevertheless, AUC values remained below 0.70, indicating modest stand-alone predictive strength despite statistical significance. Similar moderate discrimination has been reported in previous validation studies of both PPI and OPS in advanced cancer populations [6,7,8,13,20,21]. These findings are consistent with the understanding that prognostication in terminal cancer is multifactorial and unlikely to be fully captured by a single index [17,22].

The distribution of short-term mortality across combined OPS–PPI risk groups further supports this interpretation. Patients with concurrent high OPS and high PPI (OPS ≥ 3 and PPI > 6) represented the vast majority of early deaths, with markedly higher mortality within 3, 4, and 6 weeks compared with lower-risk groups. In contrast, patients with low values on both indices exhibited substantially lower early mortality. This graded pattern suggests that PPI and OPS reflect complementary dimensions of terminal decline—clinical deterioration and objective physiologic burden. Although PPI and OPS have been evaluated within the same patient cohorts in previous comparative studies [13,16], prospective evidence specifically examining their combined prognostic use in a homogeneous BSC population remains limited. The primary combined analysis focused on the dichotomized high-risk category defined by concurrent OPS ≥ 3 and PPI > 6, while all remaining patients were considered the lower-risk reference group. This approach was chosen to reduce reliance on very small individual subgroups, although subgroup-level findings should still be interpreted cautiously.

Kaplan–Meier analyses were fully concordant with both ROC and categorical mortality findings. Higher PPI and OPS categories were associated with significantly shorter median OS, and the combined OPS–PPI framework demonstrated clear stepwise separation of survival curves. Similar survival gradients have been reported individually for PPI [6,7,8,20] and OPS [12,13], whereas prospective evidence on their combined prognostic use remains limited.

Taken together, the concordance across ROC discrimination, early mortality distribution, and OS curves strengthens the internal consistency of our findings. At the same time, the incremental discriminatory gain of the combined OPS–PPI approach was modest. Pairwise DeLong comparisons showed that the combined model performed better than PPI alone, but did not significantly outperform OPS alone. Therefore, the combined approach should be interpreted as a practical risk stratification tool for identifying patients at particularly high risk of imminent death, rather than as a clearly superior predictive model. Decision curve analysis also supports the clinical relevance of the combined OPS–PPI classification as a bedside risk stratification approach. In practical terms, the observed net benefit pattern suggests that patients with both OPS ≥ 3 and PPI > 6 may represent a clinically prioritized high-risk group for short-term mortality, particularly within the 3-week and 6-week timeframes. Clinically, this may help prioritize end-of-life care planning for patients most likely to have very limited survival, while avoiding interpretation of the combined approach as a definitive individual-level mortality prediction model. The more limited findings for 4-week mortality further support a cautious interpretation of this clinical utility.

In the extended multivariable Cox regression analysis adjusted for age, sex, primary tumor site, CRP, and albumin, the combined high-risk group defined as OPS ≥ 3 and PPI > 6 remained independently associated with poorer OS. The persistence of the prognostic impact of the combined high-risk group after adjustment for inflammatory and nutritional markers, such as CRP and albumin, suggests that this combined classification may provide additional risk stratification value in terminal cancer patients. In contrast, primary tumor site was not independently associated with OS, suggesting that short-term survival in this end-of-life cohort may be driven more by global clinical and physiological deterioration than by tumor site alone [17,22,23].

Treatment options for patients with advanced cancer should be individualized according to tumor type, performance status, organ function, symptom burden, prior treatments, and patient preferences. In patients with an appropriate clinical condition, disease-directed treatments such as chemotherapy, immunotherapy, targeted therapies, or palliative radiotherapy may be considered. However, in the terminal phase, particularly in patients with ECOG performance status 3–4, marked clinical deterioration or organ failure, and no expected meaningful clinical benefit from further anticancer treatment, the main approach is BSC. In this context, BSC represents a comprehensive care approach that includes symptom control, psychosocial support, discussion of goals of care with patients and families, and avoidance of non-beneficial interventions. Early integrated palliative care should also be considered an essential component of advanced cancer care, rather than an approach limited to the end-of-life period. Evidence and current guidelines suggest that early palliative care integration may reduce symptom burden, improve quality of life, and facilitate timely discussions about goals of care [24,25]. Therefore, prognostic tools that support short-term mortality risk stratification may help structure the decision-making process when transitioning from disease-directed treatment to supportive care [25,26].

From a clinical perspective, both PPI and OPS use readily available clinical and laboratory parameters, making them suitable for bedside risk assessment in palliative care settings. Although the AUC-based incremental difference was modest and the combined classification did not consistently show greater discrimination than each individual score, the combined OPS–PPI approach may still help identify a clearly defined high-risk group after a BSC decision has been made. Patients with both OPS ≥ 3 and PPI > 6 may be considered at particularly high risk of imminent mortality. Rather than directing treatment decisions by itself, this approach may function as a structured prompt to prioritize symptom-focused care planning, timely family discussions, documentation of goals-of-care decisions, and reassessment of invasive or non-beneficial procedures. This approach is intended to complement, rather than replace, clinician judgment. When applying these findings in clinical practice, however, extrapolation to outpatient palliative care populations, hospice settings, or non-specialized care environments should be made with caution because this cohort consisted of hospitalized patients managed in a tertiary cancer center. Beyond these considerations, prognostic tools such as OPS and PPI should be regarded as supportive aids for patient-centered and empathetic communication, taking into account patient preferences, cultural context, and clinical circumstances.

## 6. Limitations

Several limitations should be acknowledged. This was a single-center study with a relatively limited sample size, conducted in a Turkish tertiary cancer center and including only hospitalized patients receiving BSC; therefore, generalizability to outpatient palliative care populations, hospice settings, or non-specialized care environments may be limited. Although all patients were managed with a BSC approach, the cohort included heterogeneous tumor types, potentially introducing biological variability.

Predefined literature-based cut-off values were used for OPS and PPI without recalibration for this specific population. In addition, comorbidities and formal frailty indices were not systematically recorded and therefore could not be included in the multivariable analysis. Although ECOG performance status and the components of PPI and OPS partially reflect functional and physiological decline, the absence of formal comorbidity and frailty assessment may have limited adjustment for baseline vulnerability.

Given the number of evaluated predictors relative to the cohort size, a potential risk of overfitting cannot be excluded, and the adjusted estimates should therefore be interpreted with caution. Formal internal validation using bootstrapping or cross-validation was not performed because of the limited sample size, the relatively small number of patients in some combined risk subgroups, and the exploratory nature of the combined OPS–PPI risk stratification approach. To reduce reliance on very small individual subgroups, the primary combined analysis classified patients with both OPS ≥ 3 and PPI > 6 as the high-risk group, whereas all remaining patients were grouped together as the lower-risk reference category. Nevertheless, the stability and reproducibility of the combined OPS–PPI risk stratification approach require validation in larger independent cohorts, including outpatient palliative care, hospice, and non-specialized care settings.

Dynamic or longitudinal prognostication could not be explored because PPI and OPS were assessed only once at baseline, and repeated score measurements during follow-up were not collected. The moderate discriminatory performance observed further underscores that prognostication in terminal cancer remains inherently complex and cannot be fully captured by structured scoring systems alone.

## 7. Conclusions

These findings highlight the clinical relevance of structured prognostic assessment in terminal cancer patients receiving BSC. Although the individual discriminatory performance of PPI and OPS was modest, their combined use may provide additional risk stratification value for identifying patients at high risk of imminent mortality. The persistence of the prognostic relevance of the combined high-risk group after adjustment for clinical covariates supports the importance of functional and physiological decline in the end-of-life setting. Further external validation in larger independent palliative care cohorts is required before the combined OPS–PPI risk stratification approach can be considered for broader clinical implementation.

## Figures and Tables

**Table 1:** Baseline characteristics, short-term mortality, and combined risk group distribution of the study population.

Variable	(*n* = 112)	%
Age (years)		
Mean ± SD	62.3 ± 12.3	—
Median (range)	61.5 (29–86)	—
Age group		
<65 years	63	56.3
≥65 years	49	43.8
Sex		
Female	46	41.1
Male	66	58.9
Primary tumor site		
Colon	23	20.5
NSCLC	19	17.0
Stomach	17	15.2
Esophagus	10	8.9
SCLC	7	6.3
Others	36	32.1
Palliative Prognostic Index (PPI)		
≤4	14	12.5
4.5–6	28	25.0
>6	70	62.5
PPI (dichotomized)		
≤6	42	37.5
>6	70	62.5
Objective Prognostic Score (OPS)		
<3	27	24.1
≥3	85	75.9
Combined risk group (OPS–PPI)		
OPS < 3 and PPI ≤ 6	18	16.1
OPS ≥ 3 and PPI ≤ 6	24	21.4
OPS < 3 and PPI > 6	9	8.0
OPS ≥ 3 and PPI > 6	61	54.5
3-week mortality		
No	60	53.6
Yes	52	46.4
4-week mortality		
No	52	46.4
Yes	60	53.6
6-week mortality		
No	40	35.7
Yes	72	64.3

Data are presented as mean ± standard deviation, median (range), or number (%). OPS, Objective Prognostic Score; PPI, Palliative Prognostic Index. NSCLC, non-small cell lung cancer; SCLC, small cell lung cancer.

**Table 2:** Discriminatory performance of OPS, PPI, and the combined OPS–PPI model for predicting short-term mortality using ROC curve analysis.

Variable	AUC (95% CI)	Sens (%)	Spec (%)	PPV	NPV	LR+	LR−	*p* Value
3-week mortality								
OPS ≥ 3	0.617 (0.514–0.721)	89.0	76.0	0.54	0.78	1.35	0.33	0.033
PPI > 6	0.617 (0.512–0.721)	75.0	62.0	0.56	0.69	1.44	0.52	0.034
Combined model (OPS ≥ 3 and PPI > 6)	0.674 (0.573–0.774)	73.6	60.7	0.62	0.73	1.87	0.44	0.002
4-week mortality								
OPS ≥ 3	0.598 (0.492–0.704)	85.0	76.0	0.60	0.67	1.29	0.43	0.074
PPI > 6	0.545 (0.437–0.652)	67.0	58.0	0.57	0.52	1.15	0.79	0.414
Combined model (OPS ≥ 3 and PPI > 6)	0.613 (0.508–0.718)	65.6	56.6	0.63	0.59	1.51	0.61	0.039
6-week mortality								
OPS ≥ 3	0.643 (0.532–0.754)	86.0	79.0	0.72	0.63	1.47	0.33	0.012
PPI > 6	0.578 (0.466–0.690)	68.0	64.0	0.69	0.45	1.28	0.68	0.174
Combined model (OPS ≥ 3 and PPI > 6)	0.651 (0.545–0.758)	65.8	63.4	0.76	0.51	1.80	0.54	0.008

AUC, area under the curve; CI, confidence interval; LR+, positive likelihood ratio; LR−, negative likelihood ratio; NPV, negative predictive value; OPS, Objective Prognostic Score; PPI, Palliative Prognostic Index; PPV, positive predictive value; Sens, sensitivity; Spec, specificity. Discriminatory performance was assessed using receiver operating characteristic (ROC) curve analysis.

**Table 3:** Comparison of short-term mortality rates according to combined OPS–PPI risk groups.

Combined Risk Group	*n*	3-Week Mortality, *n* (%)	*p* Value	4-Week Mortality, *n* (%)	*p* Value	6-Week Mortality, *n* (%)	*p* Value
OPS < 3 and PPI ≤ 6	18	5 (27.8)	0.002	8 (44.4)	0.020	8 (44.4)	0.002
OPS ≥ 3 and PPI ≤ 6	24	8 (33.3)		12 (50.0)		15 (62.5)	
OPS < 3 and PPI > 6	9	1 (11.1)		1 (11.1)		2 (22.2)	
OPS ≥ 3 and PPI > 6	61	38 (62.3)		39 (63.9)		47 (77.0)	

Data are presented as number (% within each combined risk group). Pearson chi-square test was used; *p* < 0.05 was considered statistically significant.

**Table 4:** Multivariable Cox regression analysis for overall survival.

Variable	HR (Exp[B])	95% CI	*p* Value
Combined high-risk group (OPS ≥ 3 and PPI > 6)	1.53	1.01–2.31	0.046
Age, per year	1.02	1.00–1.04	0.025
Male sex	1.37	0.90–2.09	0.140
Primary tumor site	—	—	0.205
CRP	0.999	0.997–1.001	0.369
Albumin	0.47	0.18–1.23	0.125

HR, hazard ratio; CI, confidence interval; CRP, C-reactive protein; OPS, Objective Prognostic Score; PPI, Palliative Prognostic Index. Multivariable model adjusted for age, sex, primary tumor site, CRP, and albumin.

**Table 5:** Sensitivity ROC analysis using PPI and OPS as continuous variables.

Time Point	Continuous PPI AUC (95% CI)	*p* Value	Continuous OPS AUC (95% CI)	*p* Value
3-week mortality	0.681 (0.581–0.781)	0.001	0.629 (0.527–0.732)	0.019
4-week mortality	0.599 (0.494–0.703)	0.073	0.586 (0.479–0.693)	0.117
6-week mortality	0.641 (0.538–0.744)	0.014	0.655 (0.548–0.762)	0.007

AUC, area under the curve; CI, confidence interval; OPS, Objective Prognostic Score; PPI, Palliative Prognostic Index.

**Table 6:** Cox regression sensitivity analysis using PPI and OPS as continuous variables.

Variable	Univariate HR (95% CI)	*p* Value	Multivariable HR (95% CI)	*p* Value
PPI, per 1-point increase	1.08 (1.01–1.16)	0.023	1.06 (0.98–1.15)	0.137
OPS, per 1-point increase	1.15 (1.01–1.32)	0.036	1.06 (0.90–1.25)	0.463

CI, confidence interval; HR, hazard ratio; OPS, Objective Prognostic Score; PPI, Palliative Prognostic Index. Multivariable Cox regression was adjusted for age, sex, and primary tumor site.

## Data Availability

The data underlying this article will be shared on reasonable request to the corresponding author.

## Supplementary Materials

The following supporting information can be downloaded at: https://www.mdpi.com/article/10.3390/jcm15124502/s1, Table S1. Description of Objective Prognostic Score (OPS); Table S2. Description of Palliative Prognostic Index (PPI); Table S3. Description of Palliative Performance Scale (PPS).

## Abbreviations

AUC	Area under the curve
BSC	Best supportive care
CI	Confidence interval
ECOG	Eastern Cooperative Oncology Group
HR	Hazard ratio
LDH	Lactate dehydrogenase
LR+	Positive likelihood ratio
LR−	Negative likelihood ratio
NSCLC	Non-small cell lung cancer
NPV	Negative predictive value
OPS	Objective Prognostic Score
OS	Overall survival
PPI	Palliative Prognostic Index
PPS	Palliative Performance Scale
PPV	Positive predictive value
ROC	Receiver operating characteristic
SCLC	Small cell lung cancer
SD	Standard deviation
Sens	Sensitivity
Spec	Specificity
WBC	White blood cell count
