# Supplementary material for: Prognostic Value of the Objective Prognostic Score and Palliative Prognostic Index for Short-Term Mortality in Terminal Cancer Patients Receiving Best Supportive Care: A Prospective Observational Single-Center Study
Source: J Clin Med. 2026 Jun 10;15(12):4502. doi: 10.3390/jcm15124502 (PMC13302451; doi:10.3390/jcm15124502)
Supplement: Supplementary file 1 [file jcm-15-04502-s001.zip › jcm-4329987-supplementary.pdf]

Supplementary Table 1. Description of Objective Prognostic Score (OPS)

Parameter	Assessment	Partial score
ECOG PS	4	1.0
	1-3	0.0
Anorexia	Present	1.0
Dyspnea at rest	Present	1.0
WBC	> 11,000/μL	1.0
Total Bil	> 2.0mg/dL	1.0
Cre	≥1.5mg/dL	2.0
LDH	≥502 IU/L	1.0
Anorexia was defined as taking less than five spoonfuls per meal or less than one-third of a routine meal.		

Abbreviations: ECOG PS: Eastern Cooperative Oncology Group Performance Status; WBC: white blood cells; Bil: bilirubin; Cre: creatinine; LDH: lactate dehydrogenase

Supplementary Table 2. Description of Palliative Prognostic Index (PPI)

Parameter	Assessment	Partial score
Palliative Performance Scale	10-20	4.0
	30-50	2.5
	60-100	0.0
Oral intake	Mouthfuls or less	2.5
	Reduced but more than mouthfuls	1.0
	Normal	0.0
Edema	Present	1.0
Dyspnea at rest	Present	3.5
Delirium	Present	4.0

Supplementary Table 3. Description of Palliative Performance Scale (PPS)

%	Ambulation	Activity level and disease	Activities of daily living	Oral intake	Level of consciousness

<table>
<tr><td></td><td></td><td>symptom</td><td>(ADL)</td><td></td><td></td></tr>
<tr><td>100</td><td rowspan="3">Full</td><td>Normal activity and no symptoms</td><td rowspan="4">Full</td><td rowspan="2">Normal</td><td rowspan="4">Normal</td></tr>
<tr><td>90</td><td>Normal activity with some symptoms</td></tr>
<tr><td>80</td><td>Able to do normal activity with effort</td><td rowspan="5">Normal or reduced</td></tr>
<tr><td>70</td><td rowspan="2">Reduced</td><td>Unable to do normal job or work due to some symptoms</td></tr>
<tr><td>60</td><td>Unable to do hobby or housework due to obvious symptoms</td><td>Occasional assistance needed</td><td rowspan="2">Normal or confusion</td></tr>
<tr><td>50</td><td>Mainly sit or lie</td><td rowspan="5">Unable to do any work due to severe symptoms</td><td>Considerable assistance needed</td></tr>
<tr><td>40</td><td>Mainly in bed</td><td>Mainly assistance</td><td rowspan="3">Normal or confusion or drowsy</td></tr>
<tr><td>30</td><td rowspan="3">Bed bound</td><td rowspan="3">Total care</td><td>Reduced</td></tr>
<tr><td>20</td><td>Minimal</td></tr>
<tr><td>10</td><td>Mouth care only</td><td>Drowsy or coma</td></tr>
<tr><td>0</td><td colspan="5">Death</td></tr>
</table>
